# Sledding injuries: is safety in this winter pastime overlooked? A three-year survey in South-Tyrol

**DOI:** 10.1186/1752-2897-1-5

**Published:** 2007-11-28

**Authors:** Stefano Corra, Franco De Giorgi

**Affiliations:** 1 Emergency Department, Regional Hospital, Bolzano-Bozen, South-Tyrol, Italy

## Abstract

**Background:**

Sledding is a popular traditional pastime in northern countries. However it is only rarely thought as a potentially dangerous activity even though serious injuries and deaths do occur. The purpose of this study was to calculate the incidence, the severity and the pattern of sledding-related injuries in our area, in order to set up possible preventive measures.

**Results:**

In three consecutive winter seasons (Dec.–Apr.,2002–2005). 356 patients (182 males, 174 females, mean age 26.9 years, range 2 to 81) were referred directly to our ED after a sledding injury. One patient (male, age 21 years) was transferred from a community hospital and died on the following day. Two patients (males, age 47 and 28 years) were declared dead on the scene. In the majority of the cases the accident was due to a fall and collision with the ground or a standing object. The number of injuries showed a progressive increase during the observed seasons and all deadly accidents were observed in the last season. Injuries were divided into three severity classes: minor (ISS ≤ 3), intermediate (ISS ≥ 4 < 15), severe (ISS ≥ 15). Minor and intermediate injuries were equally distributed between males and females, whereas all severe and deadly accidents occurred to male patients. Time of accident and place of accident did not affect the injury severity. A total of 386 lesions were detected. The most common diagnosis was head trauma (14,5%), followed by knee sprain (13%), ankle sprain (11.5%), and ankle/leg fracture (9%). 41 patients required hospital admission. The mean hospital length of stay was 3.9 days and 16 patients required surgery. The most common diagnosis on admission was lower limb fracture (13 patients) and head trauma (13 patients). The percentage of pediatric injuries was much lower than that reported in other studies.

**Conclusion:**

Sledding is rarely thought of as a potentially dangerous activity, but it can result in serious injury. Better public awareness of the risks of sledding injuries is required and preventive measures like the use of helmet, soft-side protections on the tracks, regular checks of the track conditions and good lightning for night sledding should be enforced.

## Background

Sledding is a popular traditional winter pastime in South Tyrol. Up to a decade ago it was practiced mainly by local residents, but recently it has been more and more advertised as tourist attraction and the participating population includes now tourists. In a recently published tourist guide book [1] over 130 sledding tracks are described in the region. These tracks are usually obtained by transforming mountain roads leading to cattle huts into well prepared sledding tracks. Some tracks are served by ski-lift facilities and few are lightened for "moonlight sledding": Few data are available worldwide about sledding injuries [2-7], and most of them are limited to pediatric accidents. To date no safety rules regulate the use of these tracks. The purpose of this study was to calculate the incidence, the severity and the pattern of sledding-related injuries in our area, in order to set up possible preventive measures.

## Methods

For every patient referred to our Emergency Department (ED) after a sledding accident in three consecutive seasons (from December, 8^th ^to April, 25^th^, 2002–2005) the following data were collected on admission: age, gender, date, time and place of the accident, previous sledding experience, type of accident (collision with other vehicles or sleds, or fall from sled), mean of transport to the ED. Data were collected independently from the hospital documentation system and used only for the purpose of this study, according to the Italian law on privacy. Ethical committee approval was not required for this study. Type of injury and Injury Severity Score (ISS), according to Baker et al. [8], were retrospectively assigned based upon ED and hospital charts. Data concerning mortality on the scene were obtained from the regional emergency call-centre. ISS was not calculated for patients dead on the scene. Injuries were divided into three severity classes: minor (ISS ≤ 3), intermediate (ISS ≥ 4 < 15), severe (ISS ≥ 15). Rescue and hospital costs were estimated from the provincial reimbursement table, based upon the ICD-9-CM classification.

## Results

During the study period 356 patients (182 males, 174 females, mean age 26,9, median age 26,5, SD 14,4, 95% CI = 3.5 – 79.5) were referred directly to our ED after a sledding injury. Two patients were declared dead on the scene and one patient (male, age 21 years) was transferred from a community hospital to our Intensive Care Unit (ICU) He sustained a devastating head trauma after collision with a snowmobile during a night ride. Cerebral death was declared on the following day and organs were donated. He was riding with a companion (male, age 28) who was declared dead on the scene. The third deadly case (male, age 47 years) happened during a night ride, too. The man lost by mistake the sledding track, ended on a steep ski slope and hit a ski lift pole at high speed. He was declared dead on the scene. All these patients were local residents with known good expertise in sledding. No one was wearing safety devices, and all accidents happened after dinner with probably intake of alcohol.

The population served by our facility is about 190,000, including an estimate of the mean tourist daily presence during the winter season. About 20% of the population belongs to the age group 0–19 years and 13% to the age group 0–14 years [9]. The total annual incidence of sledding injuries was approximately 70 per 100,000 resident. The annual incidence of pediatric injury (age class 0–14 years) was 120 per 100,000 residents (115 considering age class 0–19 years). During the study period a total of 84,999 ED visits were registered and sledding injuries represented 0,4% of all ED visits, whereas ski and snowboarding accident related visits were 3242 (3.8% of all ED visits, annual incidence 630 per 100,000 residents). The number of injuries showed a slight progressive increase during the observed seasons and all deadly accidents were observed in the last season. Minor and intermediate injuries were equally distributed between males and females, whereas all severe and deadly accidents occurred to male patients. Senior citizen showed a slightly higher number of intermediate injuries, as compared to other age groups (Table 1). However, due to the small size of this group, the data have no relevance. 225 patients came from four nearby ski resorts, whose sledding tracks are particularly popular and served by lifts facilities. We compared the mean of ISS of these four groups of patient to determine if one track could be considered more dangerous than the others, but we did not find any difference (Table 2). We also compared the mean of ISS of the injuries occurred during daylight (8 a.m. to 16 p.m.) with those that happened during night hours (17 p.m. to 7 a.m.). Time of accident did not affect the injury severity (Table 2). In the same way, previous sledding experience did not affect the injury severity However, we remark that all the deadly accidents happened during night hours. In the fatal and severe accidents patients were always expert in sledding (Table 1). A total of 386 lesions were detected (Table 3) The most common diagnosis was head trauma, followed by knee sprain, ankle sprain, and ankle/leg fracture. Eighty-nine patients were children (age group 0–14 years). The pattern of lesion in children did not differ from adults (Table 3). A higher number of minor head trauma was observed among children and its possible explanation is parents' anxiety, that leads to seek medical attention even in minor trauma. Compared to skiing and snowboarding injuries observed in our region [10], sledding injuries shows some differences. Head trauma are equally represented in the three sports (16.7% in snowboarders and 14.8% in skiers). Knee sprains are more frequent in skiing (19,9%) and sledding accidents, rather than in snowboarders (6.9%), whereas ankle sprain and leg/ankle fracture are more often observed in sledding accidents (ankle sprain 4.2% in snowboarders and 2.1% in skiers, leg/ankle fractures 1.8% in skiers and 4.5% in snowboarders). Forty-one patients required hospital admission (Table 4). The mean length of stay was 3,9 days and 16 patients required surgery. The most common diagnosis on admission was lower limb fracture (13 patients) and head trauma (13 patients). Eight patients were rescued by helicopter and 84 required ambulance transport to the ED. An estimate of the hospital costs of these sledding injuries was about 200,000 €. Hospitalized patients accounted for about 48% of the total cost. Social cost related to the invalidity of the patients were not estimated.

**Table 1:** Patients distribution among the three ISS classes.

	**ISS 1–3 (N. of pts)**	**ISS 4–14 (N. of pts)**	**ISS ≥ 15 (N. of pts)**
1° season	77	27	1
2° season	83	29	0
3° season	91	48	1 (+2)*
			
Age classes			
0–14	65	24	0
15–24	52	26	1
25–34	68	17	0 (+1)*
35–49	59	25	1 (+1)*
≥50	7	12 (p < 0.05)**	0
			
Gender			
Male	122	59	2 (+2)*
Female	129	45	0
			
Technical level			
Expert	57	17	2 (+2)*
Intermediate	121	45	0
Beginner	63	32	0

* dead on the scene.

** κ square test. Where not specified, p was not significant.

**Table 2:** Comparison of the injuriy severity between day and night and in four different resorts.

	N° of patients	ISS mean	SD	CI 95%	Range	p*
Time of injury						
Daylight	285	2.51	3.25	0.38	1–43	n.s.
Night	72	2.76	4.45	1.05	1–34	n.s.
						
Place of injury						
Reinswald	92	2.34	2.10	0.43	1–9	n.s.
Obereggen	67	2,30	2.27	0.55	1–9	n.s.
Seiseralm	38	2.84	2.77	0.91	1–9	n.s.
Rittenhorn	28	1.93	1.36	0.53	1–4	n.s.
						
Total injuries	357	2.56	3.52	0.37	1–43	

* Student's t test was used to analyze mean differences.

**Table 3:** Pattern of lesions observed

HEAD AND SPINE	Total number of lesions (%)	A.I.S.*	N. of lesions in adult population (%)	N. of lesions in pediatric population (%)
Minor head trauma (no neurological signs)	40 (10)	1	21 (7)	19 (19)
Brain concussion (nerological signs with negative CT)	16 (4)	2	13 (4.5)	3 (3)
Intracranial lesion	2 (0.5)	3(1), 5(1)	2 (0.7)	0
Facial fracture	5 (1.5)	1(3), 2(2)	4 (1)	1 (1)
Cervical sprain/vertebral contusion (without fracture)	28 (7.5)	1	21 (7)	7 (7)
Vertebral fracture	10 (3)	2(9), 3(1)	9 (3)	1 (1)
Spinal lesion	1 (<0.5)	4	1 (<0.5)	0
				
UPPER EXTREMITY				
Contusion (shoulder/arm/forearm/hand)	30 (8)	1	19 (6)	11 (11)
Shoulder dislocation	3 (1)	3	2 (0.7)	1 (1)
Clavicular fracture	3 (1)	2	1 (<0.5)	2 (2)
Humerus fracture	2 (0.5)	2(1), 3(1)	0	2 (2)
Forearm/wrist fracture	10 (3)	2(8), 3(2)	5 (2)	5 (5)
Hand/finger fracture	6 (1.5)	1	4 (1)	2 (2)
				
LOWER EXTREMITY				
Contusion (thigh/leg/foot)	21 (5.5)	1	13 (4.5)	8 (8)
Femur fracture	2 (0.5)	3	2 (0.7)	0
Leg/ankle fracture	34 (9)	2 (25), 3(9)	28 (10)	6 (6)
Knee sprain (with severe ligaments involvement)	11 (3)	2	11 (4)	0
Knee sprain (without or minor ligaments involvement)	38 (10)	1	32 (11)	6 (6)
Ankle dislocation	2 (0.5)	3	2 (0.7)	0
Ankle sprain	45 (11.5)	1	39 (13.5)	6 (6)
Foot/toe fracture	5 (1.5)	1	4 (1)	1 (1)
				
CHEST/ABDOMEN/PELVIS				
Contusion	18 (4.5)	1	15 (5)	3 (3)
Rib fracture	20 (5)	1(18), 2(2)	18 (6)	2 (2)
Hemo- and/or pneumothorax	1 (<0.5)	3	1	0
Internal abdominal injury	2 (0.5)	3	1	1 (1)
				
OPEN SKIN WOUND				
Face/head	19 (5)	1(17), 2(2)	10 (3.5)	9 (9)
Upper/lower extremity	12 (3)	1(10), 2(2)	9 (3)	3 (3)
Abdominal wall wound	1 (<0.5)	2	1	0

* A.I.S.: Abbreviated Injury Score. When two scores are applied to the same lesion, the number in parenthesis indicates the number of patients with the same score.

**Table 4:** Admitted patients.

Ward	N. of Pts	Length of stay (days), **mean **(SD, CI 95%, range)	Main admission diagnosis	N. of Pts requiring surgery
ED – Short term	14	**1.29 **(0.61, 0.35, 1–3)	Brain concussion (9) Rib fracture (2) Vertebral fracture (1) Vertebral contusion (2)	0
Orthopedics	19	**5.63 **(6.14, 2.96, 1–20)	Lower limb fracture (13) Vertebral fracture (4) Upper limb fracture (2)	13
Pediatrics	4	**3.5 **(2.08, 3.31, 1–6)	Brain concussion (3) Vertebral contusion (1)	0
Surgery	2	**5.5 **(N.A.)	Hemothorax (1) Spleen-kidney lesion (1)	1
Neurosurgery	2	**6.5 **(N.A.)	Spinal lesion (1) Vertebral fracture (1)	2
I.C.U.	1	**2**	Intracranial lesions (1)	0

## Discussion

Our findings are similar to those reported in other studies [2-7]. The pattern of lesion we observed resembles that reported by Skarbek-Boroswska et al. [6] in a large United States emergency services survey. The overall incidence of sledding injuries is in our region is higher than that reported in other studies [4,6]. This is due to the high density of sledding tracks and to the size of the participating population, made up originally only by local residents and in the last years by a growing number of tourists. However, the most impressing data of our study, compared to other findings, is the greater incidence of sledding injuries in the adult population. Other studies showed that the vast majority of sledding-related injuries resulting in ED visits occurred in patients 19 years of age or younger, with a percentage of pediatric injuries ranging from 65 to 71 of all sledding accidents [6,7]. In our study these data are reverse: only 25% of the patients are children (age 0–14, 36% considering age group 0–19). This means that development of prevention strategies, at least in our region, should not only target the pediatric population, as stated by Skarbek-Boroswska et al. [6]. Sledding is rarely thought of as a potentially dangerous activity, but it can result in serious injury. Better public awareness of the risks of sledding injuries and preventive measures are required. At present, no safety rules regulate the use of sledding tracks in our region. Our findings could offer some suggestions to improve safety in this outdoor activity. All deadly accidents were observed during "moonlight sledding". In two cases the cause was a collision with a snowmobile and in the third case the unlucky patient lost by mistake the sledding track ending on a steep ski slope and hitting a ski lift pole at high speed. Sledding tracks should not be used by vehicles other than sleds, and "moonlight sledding" should be permitted only in tracks equipped with lightning powerful enough to prevent route mistakes with poor weather condition. In most cases injury was caused by a fall from sled and subsequent collision with a standing object. Since almost all the tracks run through woods, soft side protections in sharp turns or in steep stretches should be provided to avoid collision with trees. Snow condition should be checked daily at least in those popular tracks served by ski-lift facilities, and in dangerous conditions (ice or insufficient snow) the track should be closed. The high percentage of head injury found in this and in previous studies suggests that the use of helmets should be recommended to all participants. In the same way, the higher number of lower extremity trauma, as compared to skiers and snowboarders, would suggest the use of hard boots. In the adult population it would be also interesting to study the correlation between alcohol intake and sledding injuries.

## Competing interests

The author(s) declare that they have no competing interests.

## Authors' contributions

SC conceived the study, acquired the data, drafted the manuscript and performed statistical analysis. FDG acquired the data, helped to draft the manuscript and critically revised it.

All authors read and approved the final manuscript.
